# Dynamic entropy of human blood

**DOI:** 10.1038/s41598-021-87212-z

**Published:** 2021-04-07

**Authors:** Mariusz A. Pietruszka

**Affiliations:** Faculty of Natural Sciences, Institute of Biology, Biotechnology and Environmental Protection, University of Silesia, 28 Jagiellonska St, 40032 Katowice, Poland

**Keywords:** Biological physics, Biophysics, Physiology

## Abstract

Temperature control is a process that is used by biological systems to maintain a stable internal state for survival. People have an individually variable physiological temperature of about 36.6 °C, which can be modified by many undesirable factors. Based on an analysis of a time series of extracellular ionic fluxes that were obtained using the non-invasive solute-semiconductor interface technique, I show that this extremely specific (critical) temperature is *encoded* by a local minimum in the dynamic entropy of an isolated drop of human blood. Moreover, a dynamic zeroth-order normal fluid/“superfluid” nonequilibrium phase transition, which was reflected by a spontaneous symmetry breaking that occurred in the *phase space*, was revealed. The critical scaling of the dynamic measures for the covariates such as the spectral signature and Lyapunov exponent was also determined.

## Introduction

Detecting the presence of a regularity/irregularity or chaos in the dynamic ionic fluxes of an evolving biological system is an important task that can be solved by performing detrended fluctuation analysis and subsequent analyses of the different dynamic measures (quantities). In what follows, I will show that the results of some complementary fluctuation assessment methods for the $1/f$ scaled time series (pink noise) that is generated by the extracellular ionic fluxes in living blood cells show long-range correlations—or even coherence—at a critical value of temperature, which confirms the results for the spectral signature ($\beta$) statistics that were recently obtained^1^ for human blood, which was treated as an extended dissipative dynamical system using a wider range (Kolmogorov–Sinai entropy, Hurst exponent, Lyapunov exponent, autocorrelation, average mutual information and Takens’ phase space reconstruction) of advanced statistical methods. Moreover, further experimental evidence was found for the claim that the autonomous organisation of a cell (or ensemble of cells) is accomplished by self-organised criticality, which is an orchestrated instability that occurs in a system.

After the temperature scales were established at the beginning of the eighteenth century, it was found by taking many measurements that a healthy human being has a temperature of about 36.6 °C. [Note that temperature is an intense variable, and therefore, it does not depend on the “size” of a system]. Hence, it was an *empirical* fact. Conversely, although entropy, which is an extensive variable, cannot simply be measured, it can be *calculated* and interpreted according to whether the system was classical or quantum, static or dynamic^2^. Here, it was found that by performing such calculations, the profound minimum in the dynamic entropy *vs* temperature might be localised. This local extreme indicates a temperature of about 36.6 °C, which is the main finding in this article.

The last statement means that even *without* knowing this temperature, its peculiar value can be read from a physiological time series of an *isolated* drop of human blood of a healthy individual. This can be accomplished by performing a detrended fluctuation analysis on a uniformly sampled time signal (voltage) to obtain the Hurst exponent, the largest Lyapunov exponent or dynamic entropy (defined as the “entropy rate”^2^) using numerical calculations. These results are also consistent with recently published^1^ experimental data using adequate mathematical procedures^3^ that enable these different measures to be estimated.

The history of this enterprise, which culminated in the present paper dates back to the statement that the “kinks” in the chemical potential localise the critical temperatures in the investigated condensed-matter system at the (classical) phase transitions^4–8^. It was noted that a system undergoes a phase transition at such a (critical) temperature for which the chemical potential acquires its critical value. In addition, it was found that these phase transitions can be detected by a kind of proximity effect^9,10^. Consequently, a similar “contact” method was proposed to determine the level of the chemical (redox) potential for individual cell ionic oscillations using an n-type semiconductor—electrolyte interface^11^. The last method enabled the characteristic temperatures of growing pollen tubes and peripheral human blood to be localised^1^. Bearing in mind that specific heat is proportional to a derivative of entropy and behaves similarly to the chemical potential during phase transitions^12^, the natural consequence was to study the temperature dependence for entropy and the other dynamic quantities for human blood. It should be noted that, unlike the usual concepts of entropy, the kind of entropy that is considered in this article is not a function of the *state* of a system, but rather a function of its dynamics.

## Results and discussion

Detrended fluctuation analysis, which removes the linear trends, mean value or (piecewise) linear trend^13^ from a vector of an experimental time series data^1^, was conducted after which the different dynamic measures were calculated. The Hurst exponent^14^ was calculated using an R/S analysis^15^. A corrected R/S method, an empirical and corrected empirical method and a theoretical Hurst exponent were also calculated. Estimating the Hurst exponent for a data set provided a measure of whether the physiological series was a pure random walk or whether it had underlying trends. The largest Lyapunov (${\Lambda }$) exponent^16,17^, which is by definition the rate of the exponential separation with the time of initially close trajectories and describes the speed of the convergence or divergence of trajectories in each dimension of the attractor, was also estimated. The autocorrelation function (determines the presence of a periodic signal that is obscured by noise) and the average mutual information^18^ were calculated to obtain the time delay (and consequently, the tau.acf and tau.ami variables as in Supplementary Information), which was necessary to compute the embedding dimension for maximal Lyapunov exponent and to reconstruct the phase space^19^ for the control system and the human blood (Table 1 and SI Figs. 1–3). The calculation in R programming language^3^ was performed on the detrended raw data from a number of (15 × 5000 × 2) time series (at a sampling rate of 4.1 Hz).

**Table 1:** Human blood. Multivariate analysis of the extracellular ion fluxes at a critical temperature—dynamic measures.

Temperature	Spectral signature ($\beta$)^1^	Empirical Hurst exponent [theoretical]	Largest Lyapunov exponent (emb. dims)	Approximate entropy ($S_{a}$)	Sample entropy ($S_{s}$)
36.6 ± 0.5 °C Normal	1.0067 ± 0.024	0.9941012	− 14.04 (6)	0.2584685 Low value *Deterministic*	0.1454900
Control (0.9% NaCl) 36.8 ± 0.5 °C	0.475 ± 0.025	0.8140833 [0.530]	− 15.01 (12)	0.8272206 High value *Random*	0.7133492

**Figure 1:**
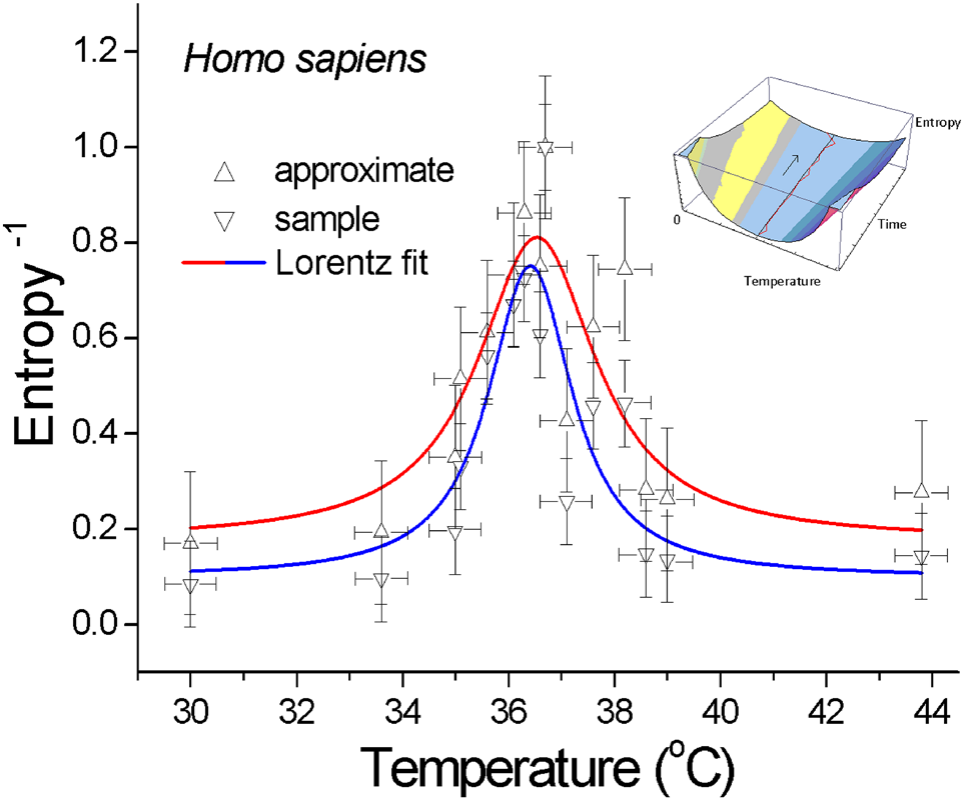
Inverse of the approximate $\left( {S_{a} } \right)$(or sample—$S_{s}$) dynamic entropy as a function of temperature for an isolated drop of human blood (normalised). The points, which were calculated from an experimental time series for the extracellular ionic fluxes, yielded the critical temperature *T*_c_ = 36.54 ± 0.07 °C (36.42 ± 0.16 °C) with a half-width of 2.67 ± 0.12 °C (1.86 ± 0.68 °C). The solid red (blue) line corresponds to the Lorentz fit with the determination coefficient *R*^2^ = 0.72 (0.75), which is smoothed by the B-spline. The temperature was measured at 20 min intervals (time series consisted of 5000 points; sampling 4.1 Hz) with an accuracy of ± 0.5 °C; the calculated *y*_error_ = 0.15 (0.09). The errors are represented by the drone-like objects in the plot. The calculation in R was performed on the detrended raw data from a number of (15 × 5000 × 2) time series. The data in the right parenthesis above was calculated for $S_{s}$. *Inset* Illustration of the “valley of entropy” and “arrow of time” (see text).

Finally, the approximate ($S_{a}$) and sample ($S_{s}$) entropy was determined in order to quantify the amount of regularity and the unpredictability of the fluctuations in a time series^20,21^. In general, a low value of the (approximate) entropy indicates that the time series is deterministic, whereas a high value indicates randomness (in some physiological signals this is the exception). The sample entropy is similar; however, it does not count self-matching, and it does not depend on the length of the time series as much. Needless to say, the fuzzy or permutation entropy can also be used as a nonlinear complexity measure for a time series^22–24^.

For the purpose of research, living organisms can be approximately treated as condensed-matter systems with thermodynamic constraints. The vital role of temperature for cell life is especially visible in the results that are presented in Table 1 (supported by SI Figs. 1–3) and Fig. 1 in which a pronounced peak in the dynamic entropy inverse ($S^{ - 1}$) at a physiological temperature of 36.6 °C occurs for the blood of a human being. Moreover, the inverse of the largest Lyapunov exponent (${\Lambda }^{ - 1}$) as a function of the temperature yields the optimum temperature *T*_c_ = 36.59 ± 0.09 °C (*R*^2^ = 0.84, half-width 1.09 ± 0.33 °C); a similar plot was obtained and is presented in SI Fig. 4. Looking at SI Fig. 4, we can see the resemblance to the $\lambda$ -type transition (cf. Figure 1.16 in Ref.^25^ or Fig. 4b in Ref.^26^), which, if it occurs, is of a dynamic origin in our case. It should be noted that the spectral signature $\beta$, which corresponds to the slope of the linear power spectral density plot, also reached a maximum at a physiological (optimal) temperature of about 36.6 °C—compare with Fig. 6 in Ref.^1^. Therefore, I suggest that for the optimal (critical) temperature of a system (where the entropy has an extreme), we can deal with a dynamic zeroth-order phase transition from a normal fluid flow (with viscosity) to a “superfluid” flow (with low or no viscosity) of the ionic fluxes with a diminished or no energy dissipation, respectively, which thereby facilitates their concerted motion or simply super-diffusion. If the first case occurs, at critical temperature of 36.6 °C, the fluid attains the “superfluid” (close to equilibrium, quasi-reversible) state and passes to (round-trip) the state of a normal fluid (irreversible) where it begins to flow according to the usual laws of hydrodynamics^27^.

The fact that a system violates the literal form of the ergodic hypothesis is an example of a spontaneous symmetry breaking. Here, the isotropic volume of the phase trajectory in a phase space is not conserved (Liouville's theorem has been violated) and decreases in $T_{c}$ (SI Fig. 3). In addition, the initial spherical O(3) symmetry at $T \ne T_{c}$ tends to the lower O(2) symmetry at $T = T_{c}$ (the isotropic phase volume in the 3D space in SI Fig. 3b is projected onto the circle in 2D in SI Fig. 3c). This indicates that a zeroth-order dynamic phase transition might occur (compare SI Fig. 2a with Fig. 7 in Ref.^28^ for a critical behaviour—where we observe a linear damping, which means that near the critical point the correlations that occur with *time* are long range). Note that, as yet undiscovered, hidden symmetries appear in the *phase space* of an evolving biological system. On the other hand, this collective mode of a system would be equivalent to the coherent (critical) state from our previous work^1^. Here, a new super-molecular order appeared, which is characterised by the occurrence of dissipative structures^28^—“certain fluctuations are amplified and give rise to a macroscopic current”. Supposedly, the resonance state at criticality can be elucidated by the kinetics of the synchronised ionic plasma oscillations (ionic plasmons) or even by the ionic pairs of fermions or charged bosons. However, the plasmons or other quasi-particles are coherent oscillations of all of the charges in a system, and this coherence can be understood exclusively in quantum terms^29^. Even though quantum theory does not account for the motion of macroscopic particles, quantum phenomena (interactions) are responsible for the creation of quasi-particles (collective excitations) that have been observed in the classical domain^30^. The possible superfluid (non-dissipative, with no affinities, no gradients of temperature or no gradients of chemical potential) ionic flow could be the macroscopic quantum state that was observed in the experiment at criticality, which was beyond the passive and active transport of ions.

### Entropy, spectral signature, Lyapunov exponent and cross-correlations

Because of the regular distribution of entropy $S\left( T \right)$, Lyapunov exponent $\Lambda$ and the previously obtained^1^ spectral signature $\beta$ as a function of temperature (they all form a Lorentz resonance curve around a critical point), the cross-correlations were estimated (SI Fig. 5). Therefore, the following can be suggested.

Because the Kolmogorov–Sinai entropy of finite classical dynamical systems is finite (Kouchnirenko's theorem), at criticality, we obtain an empirical relationship

$$ \beta \left( T \right) \propto \frac{1}{{S_{a} \left( T \right)}} \cong \frac{1}{{S_{s} \left( T \right)}} \propto - \frac{1}{\Lambda \left( T \right)} $$

which also applies in close proximity to the optimum temperature. The dynamic entropy $$S_{a,s} = S_{t} \left( T \right)$$, which is a Kolmogorov–Sinai invariant of the classical dynamical systems that is calculated in the time domain ($$t$$), is proportional to the reciprocate of the slope of the power spectral density,$$ \beta = \beta_{\omega } \left( T \right), $$ which was determined^1^ in the frequency domain ($$\omega )$$ at a given temperature ($$T)$$. In addition, the negative value of the largest Lyapunov exponent $$\left( \Lambda \right)$$ fulfils a similar dependence $$ \beta \left( T \right) \propto - \Lambda^{ - 1} \left( T \right)$$, which means that $$S\left( T \right) \propto \Lambda \left( T \right)$$ (which is in accordance with Margulis theorem). All of the above dynamic measures seem to show a critical point scaling in the temperature domain.

### Optimum cell life at the bottom of the entropy valley: arrow of time

While the main output data are presented in Table 1, the resonance-like curve(s) that are shown in Fig. 1 demonstrate the minimum dynamic entropy of human blood at the “optimum” temperature. The latter result may indicate that a “healthy” (i.e., normal) cell(s) evolves along a path of minimum entropy in accordance with the theorem of minimum entropy production^28^. This result strikingly suggests that a living system (here: a human being) is “prepared” by corresponding evolutionary mechanisms to live along the bottom of the entropy valley where it permanently experiences criticality (while struggling to preserve its relative stability) in order to reach its optimum performance—note that the “entropy valley” corresponds to the previous^1^ “ridge of criticality”. This comparison, however, enables the “arrow of time” to be determined because the *irreversible* evolution of a system—visualised as “a pile of sand” on which new grains of sand were slowly sprinkled to cause “avalanches”—and is associated with the phenomenon of self-organised criticality^31^, corresponds to a “random walk” along the ascending and broadening entropy valley (see the inset in Fig. 1). One can imagine that initially a narrow (a small temperature variance) valley is a strong attractor for a system. The wider (and shallower) valley (large variance) is no longer as “attractive” to an evolving system. The system temperature (at the bottom of the valley), which is slightly lower or higher than 36.6 °C, must be recurrently and self-consistently corrected by the evolving system (healing?) in order to work most effectively. According to our intuition, a system that is too far from this “attractor” can never return to these optimal conditions^16^—cell death occurs. This reveals the existence of a very refined tuning in the systems that are endowed with life. Thus, from a physical point of view, (cellular) life seems to be a struggle against entropy, which continues to grow over time. This assertion has been our everyday mental experience and intuition, but along with the comparison of the phenomenon of criticality and dynamic entropy parameterised by temperature, it has gained a scientific dimension.

The arrow of time is the concept that posits the asymmetry of time. It was developed in 1927 by Sir Arthur Eddington and is an unsolved general physics question. The standard answer to this question is that the arrow of time follows from the second law of thermodynamics ($\delta S \ge 0$)—that entropy is not time-reversible or is ultimately imposed by the evolution of the universe in time^32^. However, it seems that the arrow of time will appear naturally in a biological cell that is treated as an open (thermo)dynamic system.

### Multivariate analysis confirmed the 1/f criticality of “optimally” living cells: possible applications in biology and medicine

The (health) state of a specific system (here: ourselves) seems to be ultimately coded in the ion exchange^1^ of the blood cells, which can be relatively densely sampled (at 4.1 Hz) with a high degree of accuracy (~ 0.1 μV) using the solute-semiconductor interface technique^11^. Similar to the spectral signature *β*, it seems that the dynamic variables that were considered such as entropy ($S_{a,s}$), the Hurst exponent (simple, corrected R/S or empirical) or the largest Lyapunov exponent exhibit a well-defined extreme that can be localised at a critical temperature. This fact can possibly be used as an indicator of a “healthy” *vs* an “unhealthy” physiological state in biology and medicine not only on the basis of the entropy ($S$) value of the measured signal but also from the covariate measures. It seems that a directed in-depth analysis may provide tests for detecting certain pathological states such as cancer or inflammation states. When we consider the dynamic entropy of human blood in combination with the other indices in question, we can relate it to individually variable homeostasis, i.e., a condition in which a man finds himself in a state of (extended) criticality^1^. As is shown in Table 1, all of the spectral indices, the Hurst and Lyapunov exponents as well as the $S_{a,s}$ entropy can possibly become specific markers of various diseases after they have been verified in a clinical randomised research that has been conducted on a significant number of patients.

Additionally, the connection between the “valley of entropy” and the “arrow of time”, which arises as a result of criticality, could be the basic “road map of life” of various phenotypes of biological objects and, of course, for *Homo sapiens*. I suppose (see the double peaks in Figs. 4–5 in Ref.^1^) that a better measurement resolution (tenths of nano-volts) and denser sampling, especially an extremely accurate temperature measurement and stabilisation, could reveal the hyperfine structure of these dynamic measures for blood. The seemingly smooth and featureless structure of the dependence of entropy on temperature could carry rich and fundamental information about the dynamic “state” of the system under study. However, we must be aware that there might also be an alternative scenario: a universal (or individually variable) critical temperature for all humans as a body reference value for homeostasis and as a benchmark for good health.

### Emergent behaviour at criticality

A lower or higher entropy value could be associated with a different degree of energy dissipation in the longitudinal or transverse modes of the oscillations of pollen tubes^26,33^ in which the energy dispersion is considerably lower in the direction of growth. Recently we observed long-range correlations of plant and human cells at “critical” temperatures^1^. From the result of $\Lambda \left( T \right) < 0$ and the small $S_{a,s} \left( T \right)$ value at the optimum (Table 1), it can be concluded that a system oscillates in a stable manner, the influx/efflux of particles is uniform, the molecular engines work evenly and there is a low level of deterministic chaos, which indicates an emergent (collective) behaviour at criticality. Therefore, a similar conclusion concerning the intervening ions can be drawn for the pollen tubes that evolve (oscillate) in a consistent manner at criticality.

Finally, the following conclusion can be drawn from our recent and current observations, calculations and results. Normal cellular life is situated on an extended critical ridge or—equivalently—on the bottom (minimum) of an entropy valley that has a well-defined arrow of time through criticality. It seems that the classical path of least action^34^ for dynamic systems corresponds to the (most energy efficient) principle of the least dynamic entropy for a “chaotic” thermodynamic system. This assertion is in line with the theorem^35^ that the non-equilibrium system develops in such a way that it attains the minimum entropy production that also applies to complex systems in biology^36^.

## Conclusions

Entropy is a crucial state variable in thermodynamics, statistical mechanics, quantum mechanics and information processing that carries global information about whether a system is ordered, less ordered or disordered. The dynamic entropy of a living system, which is considered in this article, is not a function of the state of a system, but a function of its dynamics. It was found that the dynamic entropy of human blood as a function of temperature can be determined (calculated) by analysing the time series of the electromotive force that is generated by the unperturbed extracellular ion fluxes. Due to these critical fluctuations, the dynamic entropy of human blood has a (molecularly coded) minimum at an exceptionally specific temperature of about 36.6 °C. What is more, at a critical temperature, entropy reflects the stable deterministic (wave) component that underlies the dynamics of this system. The implications of these important observations can be far-reaching and can be of broad significance not only in physiology and medicine, but also in the basics of the biophysical sciences, which would enable “hidden treasures” to be uncovered in the nonequilibrium statistical mechanics of the phenomenon of life when it is treated as a dynamical process.

## Supplementary Information

Supplementary information.

## References

[1] Pietruszka M, Olszewska M (2020). Extracellular ionic fluxes suggest the basis for cellular life at the 1/*f* ridge of extended criticality. Eur. Biophys. J..

[2] Wehrl A (1978). General properties of entropy. Rev. Mod. Phys..

[3] R Core Team. R: a language and environment for statistical computing. R Foundation for Statistical Computing, Vienna, Austria. https://www.R-project.org/ (2020).

[4] Matlak M, Pietruszka M (1999). Chemical potential evidence for phase transitions in Fermi systems. J. Alloys Compd..

[5] Matlak M, Pietruszka M (2000). Critical behaviour of the chemical potential at phase transitions. Phys. B.

[6] Matlak M, Pietruszka M, Gosławska E, Grabiec B, Eid K (1999). On the new universal possibility to detect phase transitions in correlated electron systems. Phase Trans..

[7] Matlak M, Pietruszka M, Rówiński E (2000). Experimental method to detect phase transitions via the chemical potential. Phys. Rev. B.

[8] van der Marel D (2004). Electrons and bursting waterworks. Phys. Status Solidi (b).

[9] Matlak M, Pietruszka M (2004). Phase transitions detection by means of a contact electrode. Phys. Stat. Sol. (b).

[10] Matlak M, Molak A, Pietruszka M (2004). Chemical potential induced phase transitions. Phys. Stat. Sol..

[11] Pietruszka M, Olszewska M, Machura L, Rówiński E (2018). Single measurement detection of individual cell ionic oscillations using an n-type semiconductor—electrolyte interface. Sci. Rep..

[12] Matlak M, Pietruszka M (2001). Comparative study of the specific heat and chemical potential at phase transitions. Solid State Commun..

[13] Borchers, H. W. pracma: Practical Numerical Math Functions. R package version 2.2.9. http://CRAN.R-project.org/package=pracma (2019).

[14] Hurst HE (1951). Long-term storage capacity of reservoirs. Trans. Am. Soc. Civ. Eng..

[15] Weron R (2002). Estimating long range dependence: finite sample properties and confidence intervals. Phys. A.

[16] Wolf A, Swift JB, Swinney HL, Vastano JA (1985). Determining Lyapunov exponents from a time series. Physica.

[17] Rosenstein MT, Collins JJ, De Luca CJ (1993). A practical method for calculating largest Lyapunov exponents from small data sets. Phys. D.

[18] Shannon CE (1948). A mathematical theory of communication. Bell Syst. Tech. J..

[19] Takens, F. Detecting strange attractors in turbulence. Lect. Notes Math. 366–381 (1981).

[20] Pincus SM (1991). Approximate entropy as a measure of system complexity. Proc. Natl. Acad. Sci. USA.

[21] Hegger R, Kantz H, Schreiber T (1999). Practical implementation of nonlinear time series methods: the TISEAN package. Chaos.

[22] Li P (2019). EZ entropy: a software application for the entropy analysis of physiological time-series. BioMed Eng. OnLine.

[23] Bandt C, Pompe B (2002). Permutation entropy: a natural complexity measure for time series. Phys. Rev. Lett..

[24] Yan R, Liu Y, Gao RX (2012). Permutation entropy: a nonlinear statistical measure for status characterisation of rotary machines. Mech. Syst. Signal Process..

[25] Stanley HE (1971). Introduction to Phase Transitions and Critical Phenomena.

[26] Pietruszka M (2020). Chemical potential-induced wall state transitions in plant cell growth. J. Plant Growth Regul..

[27] Maslow VP (2004). Zeroth-order phase transitions. Math. Notes.

[28] Prigogine I (1978). Time, structure, and fluctuations. Science.

[29] Jacak JE, Jacak WA (2020). New wave-type mechanism of saltatory conduction in myelinated axons and micro-saltatory conduction in C fibres. Eur. Biophys. J..

[30] Pines D (1999). Elementary Excitations in Solids.

[31] Bak P, Tang C, Wiesenfeld K (1988). Self-organized criticality. Phys. Rev. A.

[32] Gusin P (2010). Entropy of the Universe. Postępy Fizyki.

[33] Haduch-Sendecka A, Pietruszka M, Zajdel P (2014). Power spectrum, growth velocities and cross-correlations of longitudinal and transverse oscillations of individual *Nicotiana tabacum* pollen tube. Planta.

[34] Feynman RP, Hibbs AR (1965). Quantum Mechanics and Path Integrals.

[35] Prigogine I (1947). Etude Thermodynamique des Phenomenes Irreversibles.

[36] Martyushev LM, Seleznev VD (2006). Maximum entropy production principle in physics, chemistry and biology. Phys. Rep..

