# Supplementary material for: Dynamic entropy of human blood
Source: Sci Rep. 2021 Apr 7;11:7587. doi: 10.1038/s41598-021-87212-z (PMC8026995; doi:10.1038/s41598-021-87212-z)
Supplement: Supplementary file 1 — Supplementary information. [file 41598_2021_87212_MOESM1_ESM.docx]

**Supplemental Information**

**Dynamic entropy of human blood**

Mariusz A. Pietruszka


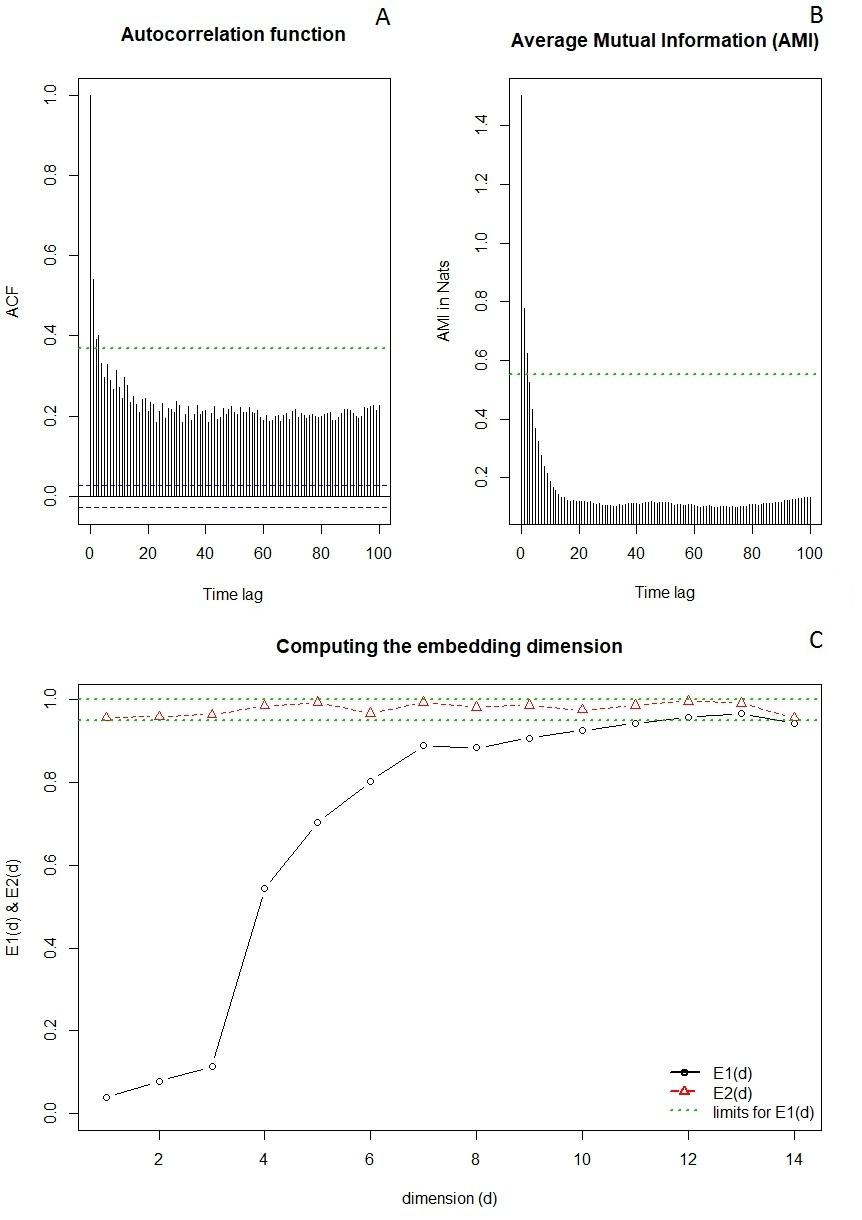


**SI Fig. 1** Results for the detrended data for control (0.9 % NaCl) **A** Autocorrelation **B** Average Mutual (Shannon) Information **C** Computing the embedding dimension (12)

**
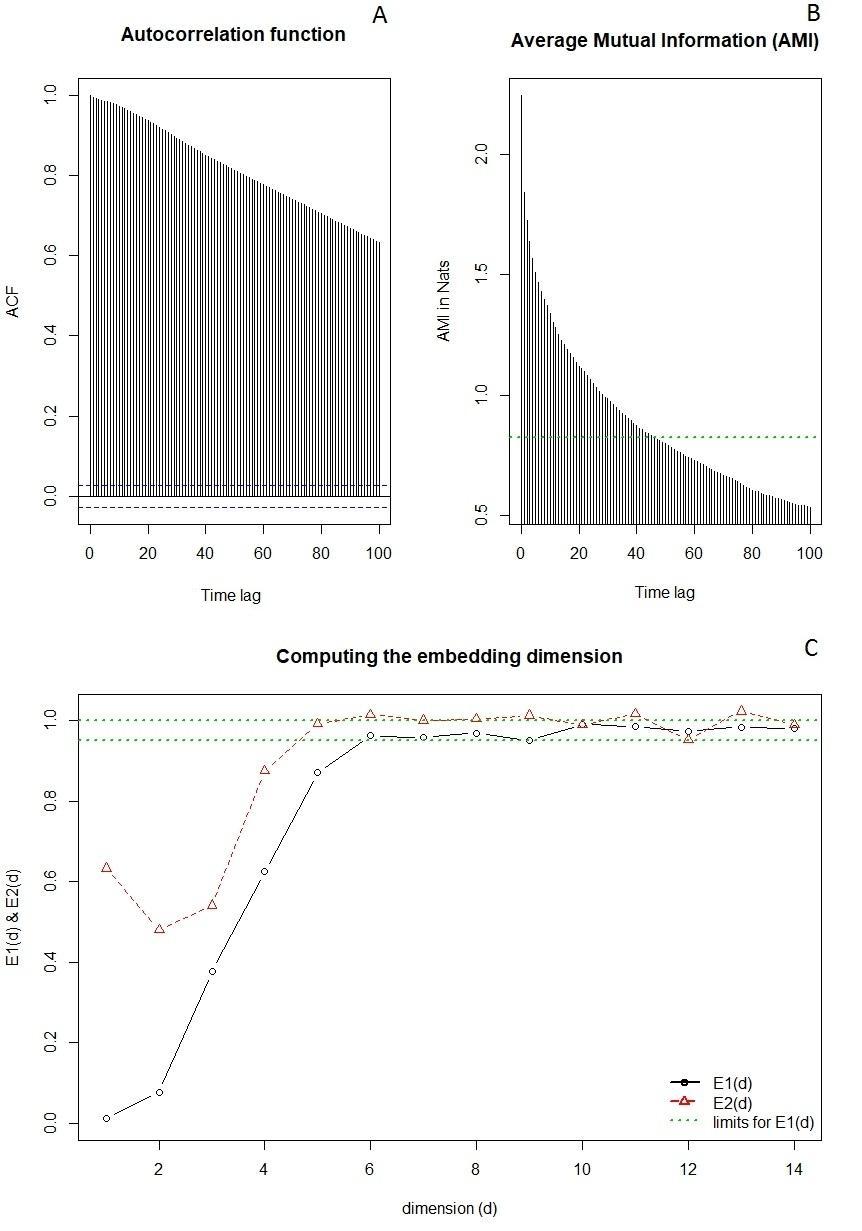
**

**SI Fig. 2** Human blood. Results for the detrended data at 36.7 °C **A** Autocorrelation **B** Average Mutual Information **C** Computing the embedding dimension (6)

**
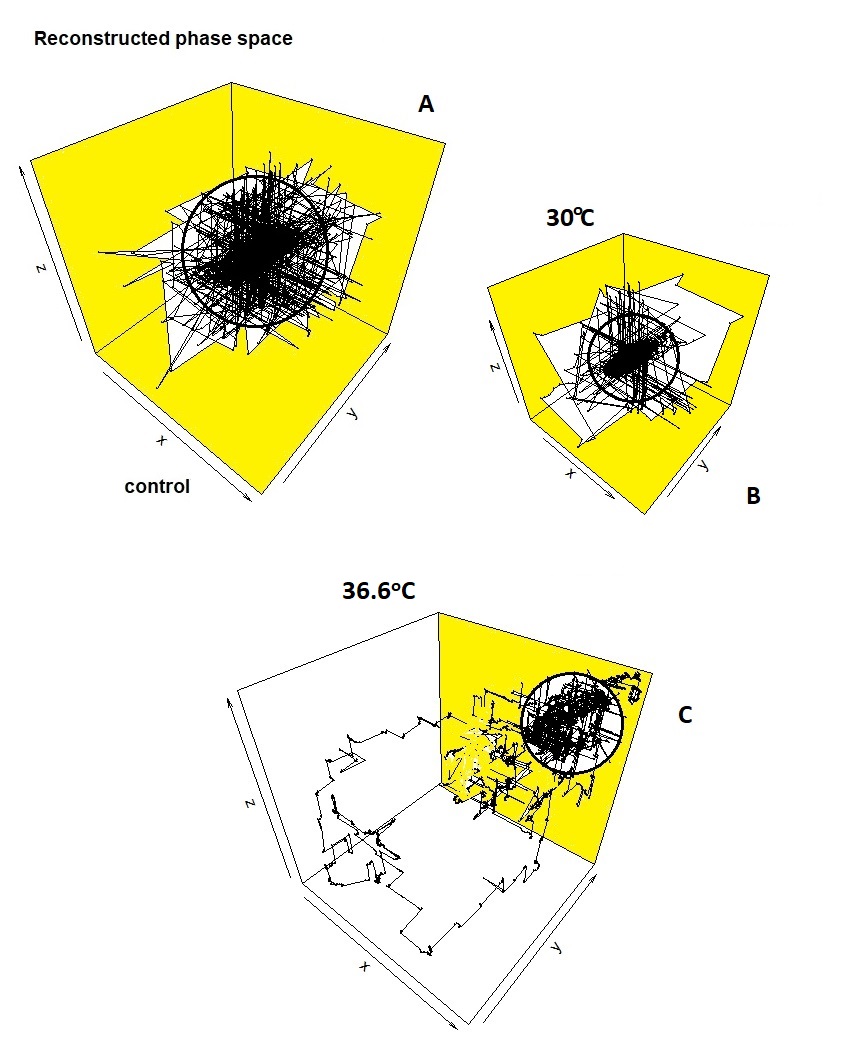
**

**SI Fig. 3** Reconstructed phase space for **A** control (0.9 % NaCl) at 36.8 ± 0.5 °C (reconstruction parameters: tau.acf = 4, tau.ami = 3) **B** human blood at 30 ± 0.5 °C (tau.acf = 4, tau.ami = 3) **C** human blood at 36.7 ± 0.5 °C (tau.acf = 4, tau.ami = 47)

**
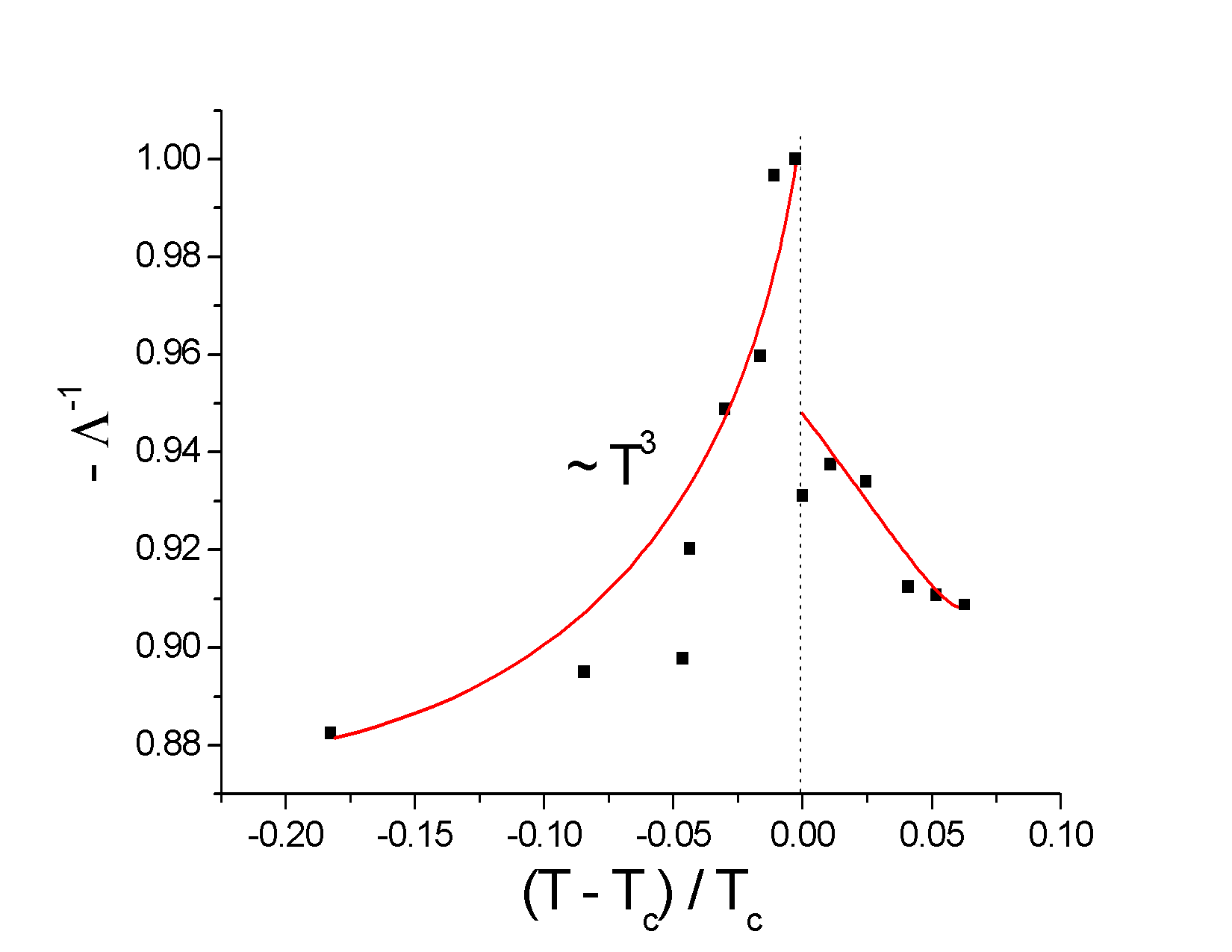
**

**SI Fig. 4** Largest Lyapunov exponent (normalised) as a function of reduced temperature for human blood. Critical temperature *T*_c_ = 36.7 °C; solid curve for *T* < *T_c_* and, separately, above *T_c_* – interpolated by cubic spline (procedure in Microcal Origin 6.0)


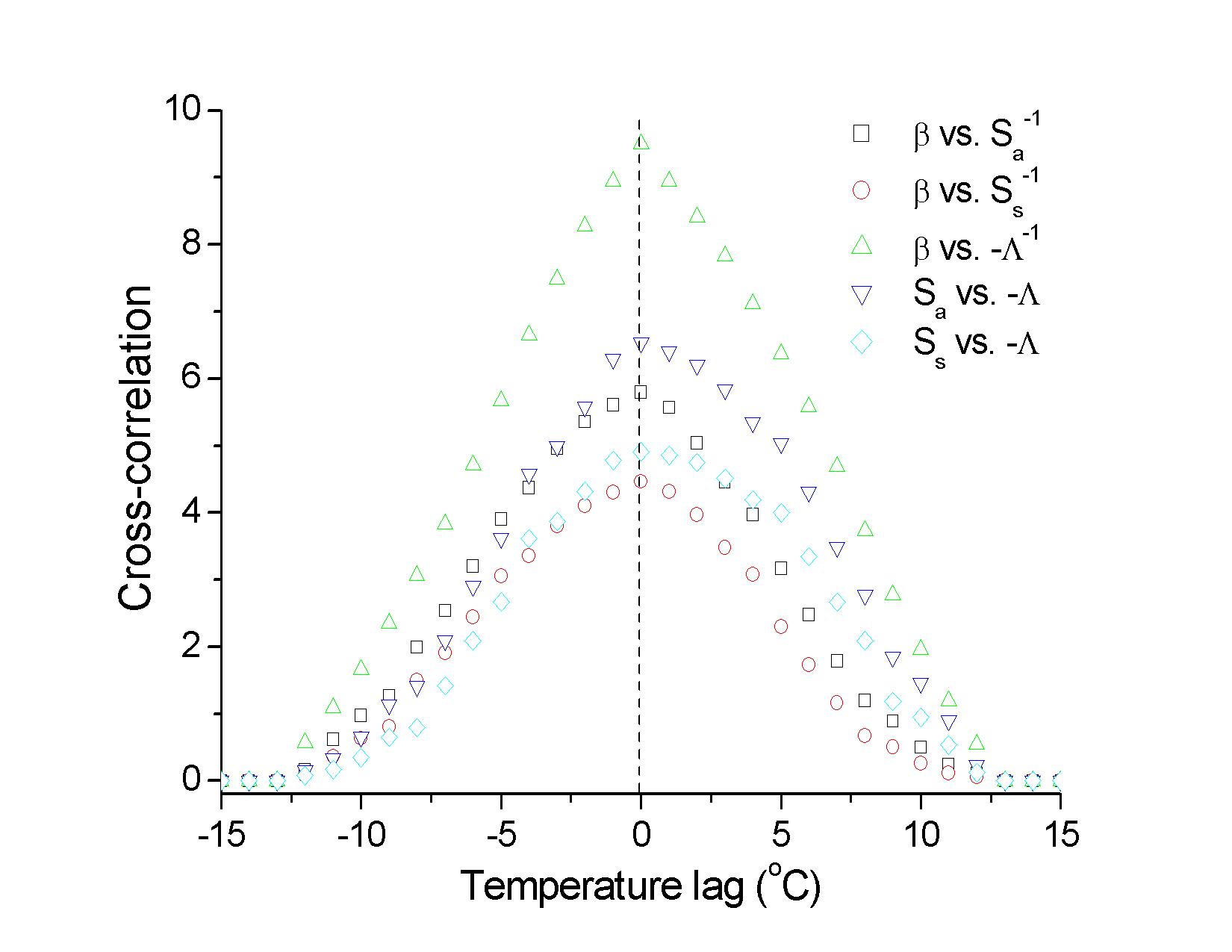


**SI Fig. 5** Cross-correlations of spectral signature (β), entropy (S) and largest Lyapunov exponent (Λ) as a function of temperature lag for human blood at about physiological temperature of 36.7 °C
